# Impact of Elexacaftor/Tezacaftor/Ivacaftor on Fat-Soluble Vitamin Status in 2 to 5 Year-Old Children Using a Cystic Fibrosis-Specific Multivitamin Formulation

**DOI:** 10.3390/children13070868

**Published:** 2026-06-29

**Authors:** Anne Munck, Jeanne Languepin, Raphael Enaud, Frederique Chedevergne, Nathalie Wizla, Natascha Remus, Marie Mittaine, Stephanie Bui, Amelie Arrouy, Megan Quinn, Amy Wahlquist, Isabelle Sermet-Gaudelus

**Affiliations:** 1 CF Center, Hospital Necker Enfants Malades, 75743 Paris, France; 2 CHU Dupuytren, CF Center, 87000 Limoges, France; 3 CF Center, CIC, Hospital Pellegrin-Enfants, 33076 Bordeaux, France; 4 Paediatric CF Center, University Hospital Lille, 59037 Lille, France; 5 Center on Rare Respiratory Diseases, Intercommunal Hospital, 94010 Creteil, France; 6 Paediatric CF Center, University Hospital des Enfants, 31059 Toulouse, France; 7 Paediatric CF Center, Hospital Pellegrin, 33076 Bordeaux, France; 8 CIC, University Hospital des Enfants, 31059 Toulouse, France; 9 Department of Biostatistics and Epidemiology, College of Public Health, East Tennessee State University, Johnson City, TN 37614, USA; 10 Center for Rural Health and Research, Department of Biostatistics and Epidemiology, College of Public Health, East Tennessee State University, Johnson City, TN 37614, USA; 11 University Paris Cite, Hospital Necker-Enfants Malades, CF Center, INSERM U1151, 75015 Paris, France

**Keywords:** Cystic Fibrosis, fat-soluble vitamins, vitamin supplementation, DEKAs Plus Liquid, elexacaftor/tezacaftor/ivacaftor (ETI), vitamin A, beta-carotene, fecal elastase-1, young children, CFTR modulators

## Abstract

**Highlights:**

**What are the main findings?**

- Serum FSV A, D and E levels remained unchanged after 12 months of ETI therapy.
- ETI therapy improved pancreatic function (FE-1) in young children with CF.

**What are the implications of the main findings?**

- The use of provitamin A (β-carotene) supports stable vitamin A levels during ETI therapy.
- Results support guidance recommendations to prioritize provitamin A in ETI-treated children.

**Abstract:**

**Background:** Young children with Cystic Fibrosis (CwCF) are at risk of fat-soluble vitamin (FSV) deficiencies due to pancreatic insufficiency, despite pancreatic enzyme supplementation. CFTR modulator therapy such as elexacaftor/tezacaftor/ivacaftor (ETI) may improve pancreatic function, but its impact on FSV levels in this age group remains unclear. We evaluated changes in FSV serum levels in a cohort transitioning to ETI, taking a CF-specific, multivitamin formulation with beta-carotene as the primary source of vitamin A (DEKAs Plus Liquid, DPL). **Methods:** Retrospective data with paired analysis from 23 CwCF (2–5 years old) were analyzed. Serum FSV levels (D, E, A) and fecal elastase-1 (FE-1) were measured at baseline and 12 months post-ETI initiation. Daily FSV doses and covariates (FE-1, naive of CFTR modulators and ETI dose) were documented. Statistical analyses included Wilcoxon signed-rank tests and general linear models. **Results:** No significant changes were observed in serum FSV levels, while FSV dosing and formulation remained unchanged. Covariates did not influence serum level changes. Interestingly, ETI significantly improved FE-1 (*p* = 0.018), with 6/18 children achieving pancreatic sufficiency (FE-1 > 200 µg/g). **Conclusions:** In young CwCF initiating ETI, FSV levels were unchanged while pancreatic function improved. The use of provitamin A (beta-carotene) as in DPL resulted in no change in vitamin A levels and aligns with consensus guidance to prioritize provitamin A in ETI-treated children. Further validation in larger cohorts is warranted.

## 1. Introduction

Children with Cystic Fibrosis (CwCF) and exocrine pancreatic insufficiency face an increased risk of fat-soluble vitamin (FSV) deficiencies (D, E, K and A) due to residual fat malabsorption despite pancreatic enzyme supplementation [1]. Cystic Fibrosis (CF) guidelines recommend routine FSV supplementation at age-appropriate daily doses [1]. We have previously reported that switching from individual vitamins to a CF-specific multivitamin formulation (DEKAs Plus Liquid, DPL) reduced vitamin deficiencies and improved both FSV levels and patient satisfaction in CwCF 0–4 years of age [2]. With the availability in France of elexacaftor/tezacaftor/ivacaftor (ETI) therapy in this age group as of the 4th quarter of 2023, its effect on FSV levels warrants evaluation. The effect of ETI on FSV levels in CF patients has revealed conflicting data [3].

We evaluated changes in serum FSV levels in this group of young CwCF who continued on DPL [2] and initiated ETI. We also considered that FSV studies in young CwCF are rare and that CF vitamin publications [4,5,6,7,8] mostly lack the detailed documentation on supplement formulation, composition and dosing that our cohort could provide. Results were evaluated against the background of possible ETI-induced improvements in fecal elastase-1 (FE-1), a marker of pancreatic (in)sufficiency.

## 2. Materials and Methods

Six CF centers in France provided retrospective anonymized data from patients included in a previous study [2], with parental consent for de-identified data sharing, resulting in 25 patients eligible to ETI based on their CF Transmembrane conductance Regulator (CFTR) variants. CwCF were aged 2–5 years when starting on ETI between July 2023 and October 2024. To assess possible changes in FSV serum levels and pancreatic function the data included routine vitamin and FE-1 measurements at baseline (M0) and 12 months (M12), after starting ETI at a dose appropriate for age/weight. Initial dosing of ETI was 1 sachet E/T/I 80/40/60 mg + 1 sachet of ivacaftor of 59.5 mg in CwCF < 6 years and <14 kg (dose A), 1 sachet E/T/I 100/50/75 mg + 1 sachet of ivacaftor of 75 mg in CwCF < 6 years and ≥14 kg (dose B). All patients were prescribed DPL (DEKAs Plus Liquid, Callion Pharma, Jonesborough, TN, USA) for their vitamin supplementation. One child was prescribed DEKAs Plus chewable tablets, which are generally equivalent to a daily dose of 2–3 mL of DPL. For this reason, daily doses of FSV are not given in ml of DPL, but as doses of FSV in International Units (IU). For the composition of DPL see Table 1.

All data such as age, height, weight, FE-1, vitamin daily doses, FSV serum levels, naive or previous use of ivacaftor or lumacaftor/ivacaftor and ETI doses were obtained from medical records. The following values at both baseline (M0) and after 12 months (M12) were used for analysis: FSV supplementation as median daily doses (IU), and serum levels of vitamin A (µmol/L), D (nmol/L), E (µmol/L) and FE-1 (µg/g stool). Prothrombin time expressed in % as a surrogate marker of vitamin K status was not analyzed as this was considered too insensitive a marker for vitamin K status. Consequently, doses of vitamin K are also not reported here. Adjustments of daily doses and treatment discontinuations were assessed quantitively. Median FSV daily doses were compared to those recommended in European Cystic Fibrosis Society (ECFS) nutritional guidelines [1]. For children > 1 year of age the recommended daily doses are: vitamin D_3_ 800–2000 IU and vitamin E 100–400 IU. There is no specific daily dose recommendation for vitamin A. Here, the daily dose must be adapted rapidly to reach the normal serum reference range [1], which happens via a feedback loop, if sufficient vitamin A is given as beta-carotene. FE-1 > 200 μg/g stool was the diagnostic criteria used for pancreatic sufficiency [1].

### Statistical Analysis

Descriptive statistics such as means, standard deviations (SD), medians, interquartile ranges (IQR), frequency counts, and percentages are reported for patient characteristics and laboratory values, as appropriate. Data is expressed in International Units (IU). Wilcoxon signed-rank tests (two-sided) were used to assess changes from M0 to M12 in paired daily vitamin doses and serum levels for children with measurements at both time points. To determine if covariates impacted the change in serum levels, general linear models were used to examine the relationships between the changes in serum vitamin levels as dependent outcome and covariates of interest (levels of FE-1, naïve or previous use of ivacaftor or lumacaftor/ivacaftor, and dose of ETI) as independent variables. Each combination of change in serum and covariate were evaluated individually. A two-sided α = 0.05 level of significance was used for all testing. All analyses and results were generated using SAS Software, Version 9.4 of the SAS System for Windows.

## 3. Results

Twenty-five (25) CwCF followed at six CF centers initiated ETI. All had been diagnosed with CF via newborn screening. During follow-up assessment, ETI was discontinued in two cases. One at M3 due to abnormal liver biological values and one at M7 due to psychological issues. Both cases were excluded from further analyses. The follow-up assessment occurred after a mean of 12.4 ± 1.6 months. The demographics and baseline characteristics of the remaining 23 patients are shown in Table 2. Eight (35%) children did not switch from another CFTR modulator, 14 (61%) switched from lumacaftor/ivacaftor, and one (4%) switched from ivacaftor. At the start of ETI therapy (M0), 11 (48%) children received dose A, and 12 (52%) received dose B. At M12, nine (39%) were receiving dose A, 13 (57%) dose B, and one (4%) was receiving half of dose A (dose was reduced at M5 due to psychological issue). Paired FE-1 values were missing for five CwCF. At M0, 16 out of 18 (80%) had severe pancreatic insufficiency (FE-1 < 100 μg/g), three out of 18 (17%) had moderate pancreatic insufficiency (FE-1 ≥ 100 μg/g and ≤200 μg/g), and one (6%) had sufficient pancreatic function (FE-1 > 200 μg/g).

Table 3 shows that the median daily dose of vitamins D, E and A did not change significantly between M0 and M12 (*p* = 0.59, *p* = 0.53, and *p* = 0.72 respectively). In contrast, FE-1 values increased significantly from M0 to M12 (*p* = 0.018). Indeed, the number of CwCF with an FE-1 above 200 μg/g stool, the threshold level for pancreatic sufficiency, rose from one at M0 to six out of 18 (33%) at M12. Table 3 also presents median serum levels of vitamins D, E, and A at M0 and M12. The 25-hydroxyvitamin D levels rose by 14% but this change was not significant (*p* = 0.12). Vitamin E and vitamin A levels did not change significantly (*p* = 0.38 and *p* = 0.52 respectively).

Table 4 presents the model results from testing whether fecal elastase levels, prior use of ivacaftor or lumacaftor/ivacaftor, and/or the dose of ETI taken by CwCF significantly influenced changes in serum vitamin D, E, or A levels between M0 and M12. The lack of significance indicates that none of these three factors had a detectable impact on the observed changes in serum vitamin levels.

## 4. Discussion

This is the first study in young CwCF (under six years of age) to evaluate ETI’s impact on serum vitamins D, E, and A, with detailed documentation of supplement composition and dosing before and after ETI initiation. This age group is of interest, as evidence suggests that younger children may experience partial restoration of pancreatic function following ETI initiation, potentially influencing the absorption and serum levels of fat-soluble vitamins [9,10,11]. Despite clinically significant improvements in pancreatic function—evidenced by increased FE-1 levels—we observed no statistically significant changes in serum vitamins D, E, or A.

Our results indicate that the DPL dose may not need to be modified for patients on ETI. Unlike previous studies on CwCF [4,5,6,7,8], which often lack detailed documentation of supplement formulation, composition, and dosing, prescribers in this study consistently recorded the vitamin formulation and daily dose used pre- and post-ETI. This detailed documentation is important for the interpretation of the results. Formulations like DPL enhance the absorption of vitamins D and A in CF and/or cholestasis [12,13,14,15,16,17]. The use of one formulation (DPL) with almost no dose changes simplifies the interpretation of serum vitamin changes post ETI.

An important element in CF vitamin supplementation is the form of vitamin A included. Many preparations contain preformed vitamin A (retinol). DPL, however, primarily uses beta-carotene, a provitamin A carotenoid, with proven safety and efficacy in CwCF [18]. The conversion of beta-carotene to vitamin A is regulated by enterocytes based on the body’s vitamin A status [19]. This mechanism prevents excessive vitamin A levels, reducing toxicity risks— a significant concern in CF, where as early as 2008, elevated serum retinol levels were reported in over 50% of preadolescent children using retinol-based supplements [8,20]. These concerns have recently resurfaced in relation to the impact of ETI therapy on vitamin A levels in CwCF.

Recent consensus-based guidance published on the nutritional management of CwCF on ETI [3] reviewed studies in CwCF over five years of age [4,5,6,7] and found no increase in vitamin E levels —a result consistent with our data. Vitamin D findings were mixed: some studies reported no change [6], while others observed modest increases of 8–18% [4,7], similar to our 14% increase. Seasonality often confounds interpretation of vitamin D data [3], but our paired measurements, taken 12 months apart, should minimize this bias.

Unlike previous studies [4,6,7], we observed no increase in serum vitamin A levels after ETI initiation, consistent with recent preliminary data [21] in 2–6-year-old CwCF. The lack of change in vitamin A levels likely results from the regulated conversion of beta-carotene (used in DPL) to vitamin A. Our findings support the consensus guidance [3] to prioritize beta-carotene to be included in supplements for children on ETI, given the increased bioavailability and toxicity risks of preformed vitamin A.

This study has several limitations. First, the sample size is small. However, this study evaluated children under six that are often underrepresented or excluded in nutritional studies in CwCF [5,8,18,22]. Confirmation of these findings with additional studies is needed. Second, FSV levels were not measured 1–3 months after ETI initiation, as required by current guidance [3]. This was not yet in place when this cohort began ETI; in France, FSV levels should now be assessed within this timeframe, alongside liver function tests for ETI monitoring. Third, dietary vitamin intake was not recorded. It is unlikely that this has influenced the outcomes, as CwCF switching to ETI are likely to maintain their daily caloric intake [23]. As for possible additional supplementation, in France, the full reimbursement of CF-related supplements likely prevents off-prescription vitamin use, as out-of-pocket costs are eliminated. This context-specific limitation may not apply in countries with different reimbursement systems.

In conclusion, this study is the first to assess the impact of ETI on serum vitamins D, E, and A in CwCF under six years of age, maintaining consistent supplement formulation (DPL), composition and dosing throughout the follow-up. Despite improved exocrine pancreatic function, ETI did not significantly alter serum vitamin levels. The use of beta-carotene as the main source of vitamin A likely contributed to stable vitamin A levels, supporting consensus recommendations to prioritize beta-carotene in ETI-treated children to mitigate vitamin A toxicity risks. As the sample size is small, further validation is needed. Together, these findings suggest that the current DPL formulation is appropriate for young CwCF on ETI.

## Figures and Tables

**Table 1:** Composition of DPL (DEKAs Plus Liquid).

Ingredient	Unit	Amount per 1 mL
Vitamin A as retinyl palmitate	IU (μg RE) *	750 (225)
Vitamin A as ß-carotene	IU (μg RE) *	5001 (1500)
Vitamin D_3_	IU (μg)	750 (18.8)
Vitamin E	IU (mg)	50 (33.6)
Vitamin K_1_	μg	500
Thiamin (B_1_)	mg	0.6
Riboflavin (B_2_)	mg	0.6
Niacin	mg	6
Vitamin B_6_	mg	0.6
Vitamin C	mg	45
Biotin	μg	15
Pantothenic Acid	mg	3
Zinc	mg	5
Selenium	μg	10

* IU = International Units, RE = all trans retinol equivalent.

**Table 2:** Patient demographics and baseline characteristics.

Characteristic	
N	23
Age (years) *	3.3 ± 1.0
Age in years (range)	2–5
Sex (Female (F), Male (M))	9 (F), 14 (M)
Length or height (cm) *	94.4 ± 8.1
Weight (kg) *	14.0 ± 2.4
Pancreatic Insufficiency (n) (%) **	16 (80%)

* Mean ± SD, ** defined as fecal elastase-1 <100 μg/g stool.

**Table 3:** Medians and interquartile ranges (IQR) of vitamin daily doses, fecal elastase-1 and vitamin serum values.

	Pairs (n)	Baseline (M0)	Month 12 (M12)	*p*-Value
Daily dose (IU)				
Vitamin D_3_ *	23	1500 (750–1875)	1500 (750–2000)	0.59
Vitamin E *	23	100 (50–125)	100 (50–125)	0.53
Vitamin A, total *	23	11,500 (5750–14,375)	11,500 (5750–14,375)	0.72
Vitamin A, as retinyl palmitate		13%	13%	
Vitamin A, as β-carotene		87%	87%	
Fecal elastase-1				
FE-1 (µg/g stool) **	18	28 (15–77)	97 (21–371)	0.018
Serum values				
Vitamin D *** (nmol/L)	22	87 (74–105)	99 (79–108)	0.12
Vitamin E (µmol/L)	23	26 (23–31)	28 (24–34)	0.38
Vitamin A (µmol/L)	23	1.3 (1.1–1.7)	1.3 (1.1–1.5)	0.52

* Recommended daily doses in European Cystic Fibrosis Society (ECFS) guidelines are given in the Materials and Methods section. ** FE-1 = fecal elastase-1, *** vitamin D = 25-hydroxy vitamin D.

**Table 4:** Impact of covariates on changes in serum vitamin levels (*p*-values).

	Overall Model Results for Covariate		
Serum Level of:	FE-1	Previous Use of CFTR * Modulator	Dose of ETI **
	β (SE), *p*-Value	Δ *** (95% CI), *p*-Value	Δ **** (95% CI), *p*-Value
Vitamin D	−0.06 (0.07) *p* = 0.42	^a^ 7.05 (−11.34, 25.44) ^b^ −2.00 (−44.78, 40.48) *p* = 0.69	−2.41 (−19.23, 14.41) *p* = 0.77
Vitamin E	−0.01 (0.03) *p* = 0.71	^a^ 3.42 (−4.95, 11.80) ^b^ −2.86 (−22.91, 17.18) *p* = 0.61	4.24 (−3.39, 11.87) *p* = 0.26
Vitamin A	<0.00 (0.00) *p* = 0.89	^a^ 0.22 (−0.33, 0.76) ^b^ −0.63 (−1.94, 0.69) *p* = 0.34	−0.26 (−0.78, 0.26) *p* = 0.31

* CFTR = CF Transmembrane conductance Regulator, ** ETI = elexacaftor/tezacaftor/ivacaftor, *** least squares mean differences between prior use of ^a^ ORKAMBI or ^b^ KALIDECO vs. no prior use, **** least squares mean difference between A and B doses of ETI.

## Data Availability

The data presented are unavailable in their original form as they originate from patient records stored in the participating centers. Most data are included in the article material. Further inquiries can be directed to the corresponding author.

## Abbreviations

The following abbreviations are used in this manuscript:

CF	Cystic Fibrosis
CFTR	CF Transmembrane conductance Regulator
CwCF	Children with Cystic Fibrosis
DPL	DEKAs Plus Liquid
ECFS	European Cystic Fibrosis Society
ETI	Fixed combination of Elexacaftor, Tezacaftor and Ivacaftor
F	Female
FE-1	Fecal elastase-1
FSV	Fat-Soluble Vitamins
IQR	Inter Quartile Range
IU	International Unit(s)
M	Male
M0	Baseline, Month 0
M12	Month 12 after baseline
